# Association of Severity and Prognosis With Elevated Homocysteine Levels in Patients With Intracerebral Hemorrhage

**DOI:** 10.3389/fneur.2020.571585

**Published:** 2020-10-19

**Authors:** Dandan Wang, Wenjuan Wang, Anxin Wang, Xingquan Zhao

**Affiliations:** ^1^Department of Neurology, Beijing Tiantan Hospital, Capital Medical University, Beijing, China; ^2^National Center for Clinical Research of Nervous System Diseases, Beijing, China; ^3^Center of Stroke, Beijing Institute for Brain Disorders, Beijing, China; ^4^Beijing Key Laboratory of Translational Medicine for Cerebrovascular Disease, Beijing, China

**Keywords:** intracerebral hemorrhage, homocysteine, prognosis, survival rate, severity

## Abstract

**Background:** Intracerebral hemorrhage (ICH) has high mortality and morbidity rates in the world. Homocysteine (Hcy) has been demonstrated to be an independent risk factor and could predict the prognosis and recurrence of ischemic stroke. In our study, we aimed to find out the relationship between Hcy levels and the severity and prognosis of patients with ICH.

**Methods:** Patients' basic characteristics and laboratory examination results, including the concentration of homocysteine, were taken at baseline from January 2014 to September 2016, and a 1 year follow-up, including the modified Rankin Scale and living status, was taken for all the patients. Logistic regression and Kaplan–Meier survival method were used to analyze the relationship between different Hcy levels and clinical outcome.

**Results:** A total of 551 patients with acute ICH from 13 hospitals in Beijing were enrolled in our study. High Hcy was detected in 284 patients (51.5%). Percentage of male, smoking, drinking, and concentration of hemoglobin A1c and triglyceride levels showed a significant difference between different Hcy level groups (all *P*-values <0.05). In the logistic regression analysis, high Hcy level is an independent risk factor for the prevalence of 3 month poor prognosis [odd ratio (95% confidence interval) = 1.601 (1.063–2.412), *P* = 0.0242], especially in female subgroup. In the 1 year follow-up period, high Hcy level patients had a significantly higher rate of fatal incidence compared to normal Hcy level patients (*P* = 0.0023).

**Conclusions:** High Hcy level was independently associated with poorer 3 month prognosis and a lower survival rate within 1 year in patients with ICH.

## Introduction

Acute spontaneous intracerebral hemorrhage (ICH) is one of the most common critical diseases in the neurological field. The Global Burden of Disease Study 2016 and Stroke in China section reported that ICH has a higher mortality rate than ischemic stroke in the world and higher morbidity rate in China than other high-income countries (1–3). However, due to the limited therapeutic methods and poor prognosis of ICH, studies on ICH prevention and therapy are still needed by now (4, 5). Homocysteine (Hcy) is a non-essential amino acid, which is largely affected by the methylenetetrahydrofolate, vitamin, and folate status. Previous studies have demonstrated that elevated total homocysteine level is an independent risk factor of ischemic stroke, and high Hcy levels predicted poorer prognosis and higher recurrence (6–8). However, few studies focus on patients with ICH and its pathogenesis in ICH is still unclear. Some inferred the atherosclerotic effect may also play an important role (9). In our study, we aimed to find out the relationship between Hcy levels and the severity and prognosis of patients with ICH.

## Materials and Methods

### Study Design and Population

The study was a prospective, multicenter, consecutive, observational cohort study conducted in 13 hospitals in Beijing from January 2014 to September 2016. All the centers were given a unified and standard training about the questionnaire collection, testing methods of laboratory indexes, and the interpretation of ICH at the beginning of the study, and regular inspections were done to ensure the quality of the study. All the images were collected by our study group and re-analyzed at the end of the study. The study protocol conforms to the ethical guidelines of the 1975 Declaration of Helsinki, as reflected in *a priori* approval by the institution's human research committee. The study was also approved by the Institutional Review Board (IRB) of Beijing Tiantan Hospital, Capital Medical University. Written informed consents were obtained from all patients or their relatives. The patients were also informed of abnormal findings and recommended treatments.

The inclusion criteria include the following: (1) ICH was diagnosis by the WHO standard and confirmed by the hospital's computerized tomography (CT) scan (10); (2) age ≥18 years old; (3) arriving at hospital within 72 h after onset; (4) first ever acute-onset ICH; and (5) written informed consent was obtained. The exclusion criteria include the following: (1) past history of ICH; (2) congenital or acquired coagulation disorders; and (3) complicated with major comorbidities or late-stage diseases. A total of 1,964 patients were enrolled and 640 of them had their Hcy concentration tested and were enrolled in the Hcy subgroup at baseline. We excluded one patient with invalid records of Hcy concentration and 88 patients without follow-up records. Finally, 551 patients were enrolled in our study.

### Assessment of Hcy Level and Other Laboratory Examinations

Superficial venous blood was collected for measurement. Serum homocysteine was assessed by enzyme kinetic method in the next morning after admission (at least 8 h without any food or intravenous infusions containing carbohydrates) in each qualified hospital. A concentration of higher than 15 mmol/l is defined as high Hcy (HHcy) in the study (11).

Other laboratory examinations, including fasting blood glucose (FBG), hemoglobin A1c (HbA1c), total cholesterol (TC), triglyceride (TG), low-density lipoprotein cholesterol (LDL-C), high-density lipoprotein cholesterol (HDL-C), and high-sensitivity C-reactive protein (Hs-CRP), were also collected during admission.

### Assessment of Epidemiological Information, ICH Condition, and Complicating Diseases

Every patient answered a standardized questionnaire (age, sex, medical history, and other basic information) administered by our trained investigators. Smoking was defined as at least one cigarette per day for more than a year. Alcohol consumption was defined as an intake of at least 80 g of liquor a day for more than 1 year. Smoking or drinking cessation was considered only if it lasted for at least 1 year. Body weight (to the nearest 0.1 kg) and body height (to the nearest 0.1 cm) were measured, and the body mass index (BMI) was calculated as body weight (kg) divided by the square of height (m^2^).

The location, hematoma volume, and etiology of ICH were recorded during patients' hospitalization. Etiology was classified to hypertension, cerebral amyloid angiopathy (CAA), secondary, and others. The secondary etiology included aneurysm, arteriovenous malformation, arteriovenous fistula, cavernous hemangioma, venous malformations, telangiectasia, venous sinus thrombosis, moyamoya disease, and coagulation disorders. Any ICH caused by trauma or neoplasm was not included in our study. We also measured the National Institute of Health Stroke Scale (NIHSS), Glasgow Coma Scale (GCS), and modified Rankin Scale (mRS) at the time of arrival at the hospital and discharge from hospital and mRS at 1 month, 3 months, and 1 year during the follow-up period for each patient separately.

Hypertension was defined as a self-reported history, taking antihypertensive medication, or a systolic blood pressure ≥140 mmHg or a diastolic blood pressure of ≥90 mmHg at baseline. Diabetes mellitus was defined as a self-reported history, current treatment with insulin or oral hypoglycemic agents, or fasting blood glucose level ≥7.0 mmol/l at baseline. Dyslipidemia was defined as a self-reported history, current use of cholesterol-lowering medicine, or a total cholesterol level ≥6.22 mmol/l or triglyceride ≥2.26 mmol/l, or low-density lipoprotein ≥4.14 mmol/l at baseline. Deep vein thrombosis was diagnosed by checking patient's lower extremities using the color venous Doppler ultrasonography. Pulmonary infection was diagnosed by the chest CT or X-ray and atrial fibrillation was diagnosed by electrocardiogram.

### Follow-Up and Outcome Assessment

A face-to-face interview at discharge and telephone interviews at 1 month, 3 months, and 1 year separately after ICH were required for all the patients. Trained research coordinators, who were blinded to patients' baseline characteristics, assessed the scores of mRS based on the functional status reported by the patients or their relatives or caregivers at each follow-up. Poor functional outcome or prognosis, namely, death or disability, was defined as a score of 3–6 on mRS. A score of 6, which indicates death, was recorded in detail, including date and etiology of death, and analyzed separately.

### Data Management and Statistical Analysis

The data management system is the SAS software (version 9.3; SAS Institute, Cary, North Carolina, USA). All the continuous variables have been checked for the normality test, and all resulted as abnormal distribution. Descriptive statistics include quartile for continuous variables and percentage of total for categorical variables. Chi-square test was used for comparison of categorical variables and Kruskal–Wallis test was used for continuous variables. Logistic regression and Kaplan–Meier survival method were used to analyze the relationship between different Hcy levels and clinical outcome. The null hypothesis was rejected for *P* < 0.05.

## Results

Out of the study population of 551 patients, there were 386 males and 165 females. All the patients were Asian, and most of them were ethnic Han (*n* = 532). There were 284 (51.5%) patients with HHcy. The baseline characteristics of the patients are shown in Table 1. Patients with HHcy had a higher percentage of male and smoking and drinking habits and a lower percentage of diabetes mellitus and blood concentration of HbA1c and triglyceride (all *P*-values < 0.05). Other laboratory results, complicating diseases, and whether there was a surgery during hospitalization had no significant difference between groups. The basic characteristics between included and excluded patients from our study population are shown in Supplementary Table 1.

**Table 1:** Baseline characteristics and their univariate association with different Hcy levels.

	**HHcy *n* = 284**	**Normal Hcy*n* = 267**	***P*-value**
Male [no. (%)]	221 (77.8)	165 (61.8)	<0.0001
Age (years; IQR)	58.0 (47.0, 67.0)	57.0 (50.0, 65.0)	0.6970
BMI (IQR)	25.4 (23.0, 27.7)	24.8 (22.5, 27.3)	0.1337
Smoking [no. (%)]	113 (39.8)	71 (26.6)	0.0011
Drinking [no. (%)]	138 (48.6)	102 (38.2)	0.0161
Previous mRS (IQR)	0 (0, 0)	0 (0, 0)	0.8038
SBP (mmHg; IQR)	160.0 (144.5, 177.0)	160.0 (142.0, 180.0)	0.9370
DBP (mmHg; IQR)	94.0 (80.0, 105.5)	91.0 (80.0, 103.0)	0.3347
FBG (mmol/l; IQR)	5.4 (4.6, 6.5)	5.6 (4.7, 7.0)	0.1231
HbA1c [no. (%)]	5.5 (5.2, 6.0)	5.7 (5.3, 6.4)	0.0008
TC (mmol/l; IQR)	4.6 (4.0, 5.4)	4.5 (3.8, 5.3)	0.2087
TG (mmol/l; IQR)	1.4 (1.0, 1.8)	1.2 (0.8, 1.6)	0.0006
LDL-C (mmol/l; IQR)	2.9 (2.3, 3.6)	2.9 (2.2, 3.4)	0.2020
HDL-C (mmol/l; IQR)	1.2 (1.0, 1.5)	1.2 (1.0, 1.5)	0.6848
Hs-CRP (mg/l; IQR)	4.5 (1.9, 12.0)	5.4 (1.8, 11.5)	0.5170
**Complicating disease [no. (%)]**			
Hypertension	188 (66.2)	185 (69.3)	0.4664
DM	32 (11.3)	55 (20.6)	0.0033
Dyslipidemia	104 (36.6)	100 (37.5)	0.8602
PI	62 (21.8)	60 (22.5)	0.9183
DVT	22 (7.8)	33 (12.4)	0.0875
AF	5 (1.8)	5 (1.9)	1.000
Surgery [no. (%)]	25 (8.8)	33 (12.4)	0.2112

IQR, interquartile range; BMI, body mass index; mRS, modified Rankin Scale; SBP, systolic blood pressure; DBP, diastolic blood pressure; FBG, fasting blood glucose; HbA1c, hemoglobin A1c; TC, total cholesterol; TG, triglyceride; LDL-C, low-density lipoprotein cholesterol; HDL-C, high-density lipoprotein cholesterol; Hs-CRP, high-sensitivity C-reactive protein; DM, diabetes mellitus; PI, pulmonary infection; DVT, deep vein thrombosis; AF, atrial fibrillation.

In the second step, we compared the ICH characteristics and severity for patients with different Hcy levels, and there is no difference for the hematoma location, volume, etiology, or severity during hospitalization between the HHcy group and normal Hcy group (Table 2).

**Table 2:** Association with ICH characteristics and severity between different Hcy level groups.

	**HHcy**	**Normal Hcy**	***P*-value**
Location [no. (%)]			0.4539
Lobar	47 (17.2)	54 (21.0)	
Deep	201 (73.6)	175 (68.1)	
Lobar + deep	4 (1.5)	7 (2.7)	
Ventricle	21 (7.7)	21 (8.2)	
Etiology [no. (%)]			0.1107
Hypertension	240 (84.5)	205 (76.8)	
CAA	6 (2.1)	9 (3.4)	
Secondary	21 (7.4)	34 (12.7)	
Others	17 (6.0)	19 (7.1)	
Break into ventricle [no. (%)]	84 (29.6)	74 (27.7)	0.6387
Break into subarachnoid [no. (%)]	29 (10.2)	28 (10.5)	1.0000
Hematoma volume (ml; IQR)	12.0 (5.0, 25.0)	12.0 (4.5, 29.7)	0.8947
GCS at first admission (IQR)	15.0 (13.0, 15.0)	15.0 (13.0, 15.0)	0.5687
NIHSS at first admission (IQR)	8.0 (2.0, 13.0)	7.0 (3.0, 13.0)	0.9878

IQR, interquartile range; CAA, cerebral amyloid angiopathy; GCS, Glasgow Coma Scale; NIHSS, National Institute of Health Stroke Scale.

**Figure 1:**
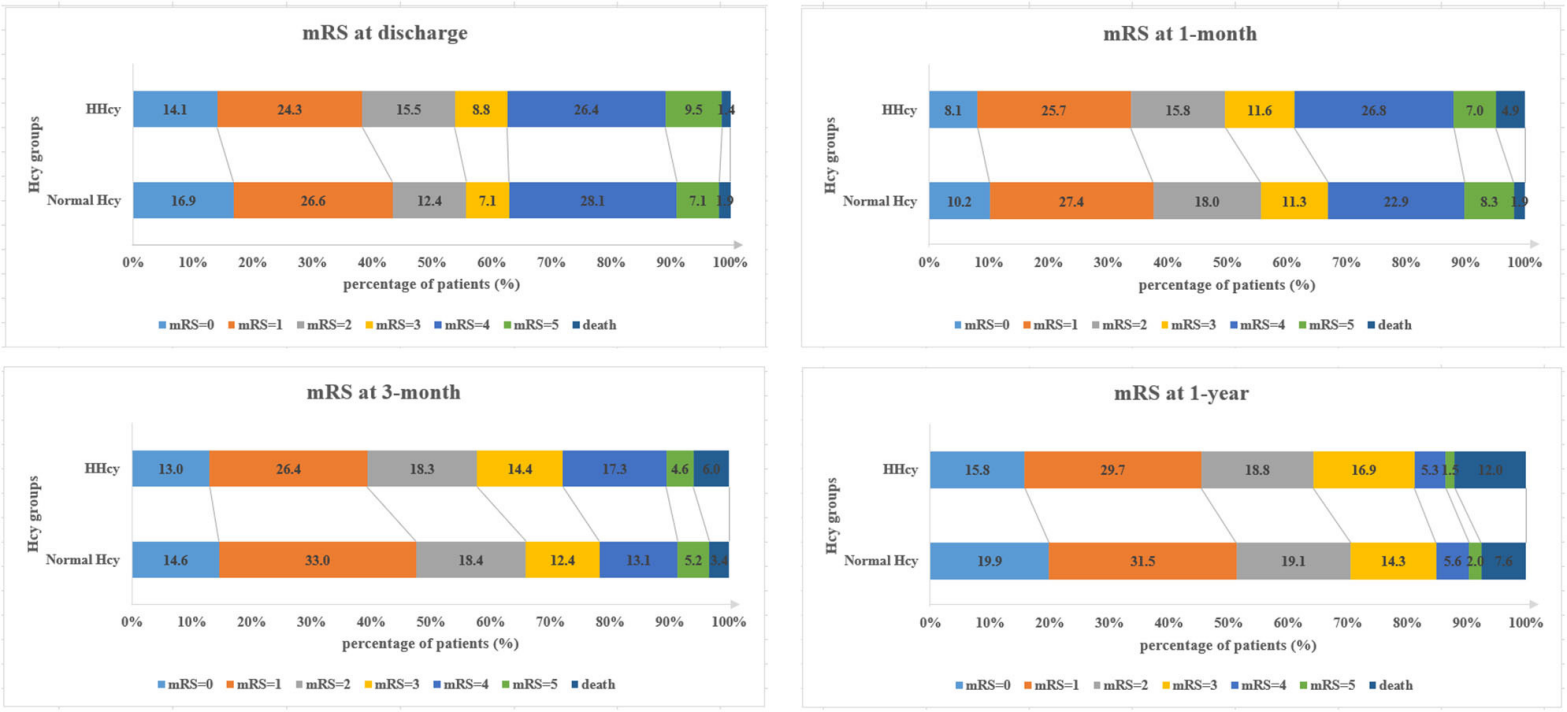
Distribution of modified Rankin Scale (mRS) in different homocysteine (Hcy) level groups.

**Figure 2:**
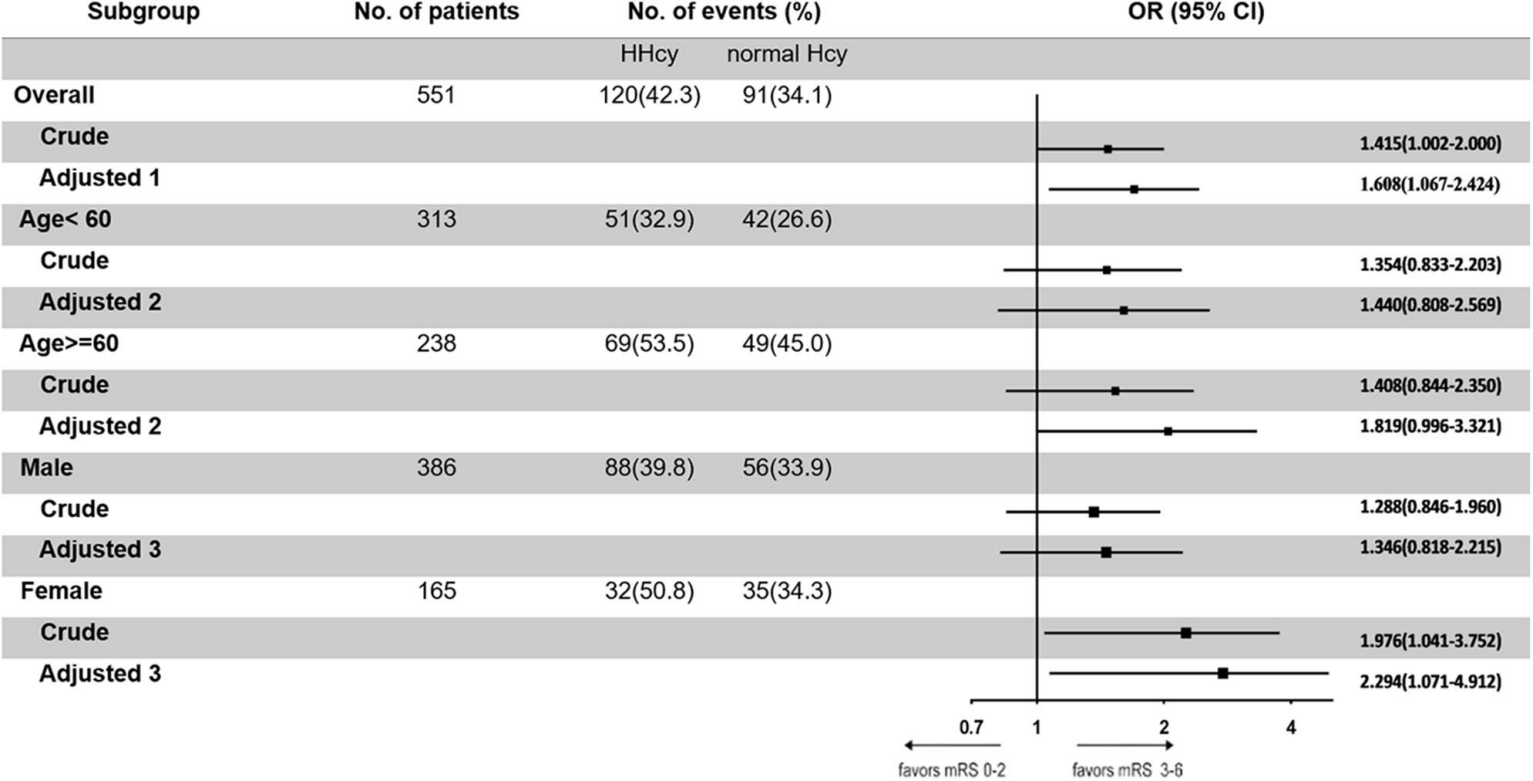
Odd ratios of homocysteine (Hcy) levels for the presence of poor prognosis at 3 months. Adjusted 1: adjusted by sex, age, body mass index, smoking, drinking, hypertension, diabetes mellitus, dyslipidemia, pulmonary infection, deep vein thrombosis, and atrial fibrillation. Adjusted 2: adjusted by sex, body mass index, smoking, drinking, hypertension, diabetes mellitus, dyslipidemia, pulmonary infection, deep vein thrombosis, and atrial fibrillation. Adjusted 3: adjusted by age, body mass index, smoking, drinking, hypertension, diabetes mellitus, dyslipidemia, pulmonary infection, deep vein thrombosis, and atrial fibrillation.

**Figure 3:**
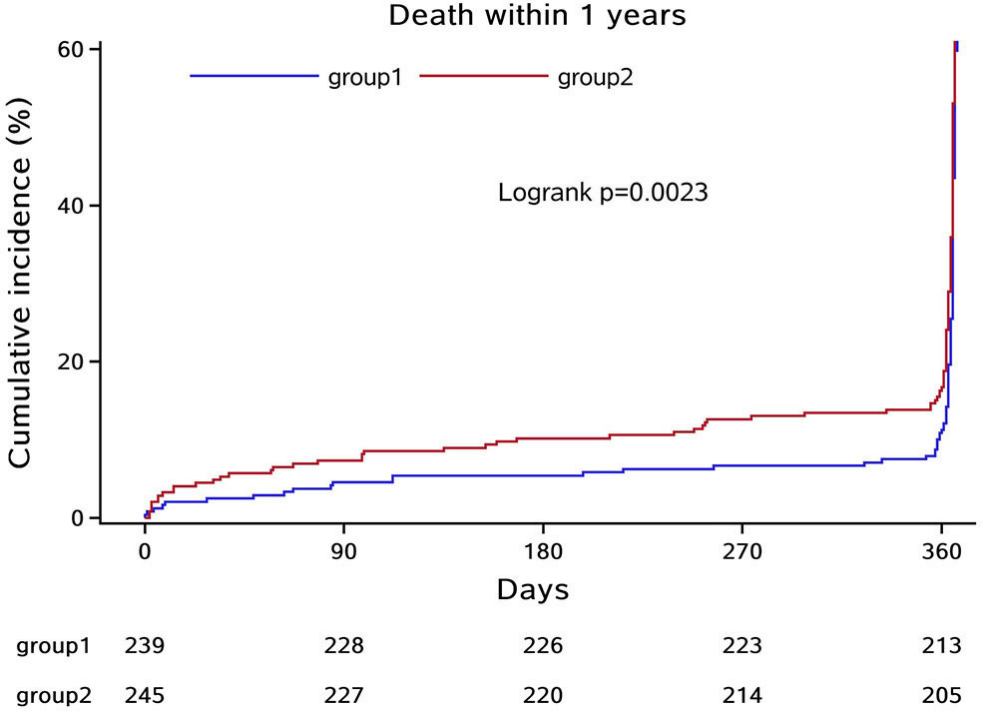
Kaplan–Meier curve of different Hcy levels for survival. Group 1: normal Hcy group; group 2: high Hcy group.

In the third step, we mainly analyzed the association between Hcy levels and prognosis of patients with ICH. For the functional outcome of mRS, the distribution of mRS is shown in Figure 1 for different Hcy level groups. No difference was shown on discharge, 1 month, and 1 year follow-up time-points between these groups, while at 3 month follow-up, patients with HHcy had a significantly higher mRS than patients with normal Hcy (*P* < 0.05, Table 3). In the logistic regression analysis, HHcy is an independent risk factor for the prevalence of 3 month poor prognosis [odd ratio (OR) [95% confidence interval (CI)] = 1.415 (1.002–2.000), *P* = 0.0490 for high Hcy level and OR (95%CI) = 1.601 (1.063–2.412), *P* = 0.0242 for high Hcy level after adjusting relevant risk factors]. In the subgroup analysis, a relationship exists especially in the female patients' group [OR (95%CI) = 2.343 (1.099–4.995), *P* = 0.0275 after adjusting relevant risk factors] (Figure 2). In the 1 year follow-up period, we recorded the date of death if the patient died due to any ICH-related or unrelated reason, and a cumulative incidence was defined as a total fatal incidence. A Kaplan–Meier curve of different Hcy levels for survival was made. The HHcy patients had a significantly higher rate of cumulative incidence compared to normal Hcy patients (*P* = 0.0023) (Figure 3).

**Table 3:** Association with ICH prognosis between different Hcy level groups.

	**HHcy**	**Normal Hcy**	***P*-value**
GCS at discharge (IQR)	15.0 (14.0, 15.0)	15.0 (14.0, 15.0)	0.6314
NIHSS at discharge (IQR)	4.0 (1.0, 10.0)	4.0 (1.0, 8.0)	0.2956
mRS at discharge (IQR)	2.0 (1.0, 4.0)	2.0 (1.0, 4.0)	0.3747
Death within hospital [no. (%)]	4 (1.4)	5 (1.9)	0.7453
mRS at 1 month (IQR)	3.0 (1.0, 4.0)	2.0 (1.0, 3.0)	0.1434
Death at 1 month [no. (%)]	13 (4.6)	5 (1.9)	0.0937
mRS at 3 months (IQR)	2.0 (1.0, 4.0)	2.0 (1.0, 3.0)	0.0474
Death at 3 months [no. (%)]	17 (6.0)	9 (3.4)	0.1638
mRS at 1 year (IQR)	2.0 (1.0, 3.0)	1.0 (1.0, 3.0)	0.0815
Death at 1 year [no. (%)]	32 (12.0)	19 (7.6)	0.1047

IQR, interquartile range; GCS, Glasgow Coma Scale; NIHSS, National Institute of Health Stroke Scale; mRS, modified Rankin Scale.

## Discussion

The current study demonstrated that HHcy level was independently associated with poorer 3 month prognosis in patients with ICH, especially in female patients. A Kaplan–Meier survival curve demonstrated that HHcy level was also associated with a lower survival rate within 1 year after ICH incident happened. These results suggest the possibility that in patients with acute ICH, high Hcy concentration plays a role in the development and recovery during their life after the disease onset.

To evaluate the severity and prognosis of patients with ICH, many indexes and scores came out in recent years. Dai et al. (12) analyzed 1,516 patients with ICH and found out there was an inverse association between GCS level and Hcy concentration. Zhou et al. (13) reported that HHcy was associated with larger hematoma volume in patients with thalamoganglionic ICH, but it might not be a predictor of the 6 month clinical outcome in patients with ICH. A meta-analysis showed that high Hcy level is positively associated with high risk of ICH and no race-specials were shown between Asians and Caucasians (9). In our study, there was no significant difference in hematoma volume, GCS, and NIHSS scores at admission between groups with different Hcy levels, but HHcy did show a poor influence on the long-time prognosis of patients with ICH, which implies that Hcy may play a long role in the development after ICH onset. Our results are consistent with most studies, but since we have different enrolled patients and adjusted factors when finding the relationship between Hcy level and outcome of patients with ICH, a contradictory result was found between our study and Zhou's study. For the cardiovascular risk factors analysis, previous studies have demonstrated that Hcy concentration is associated with the disease of hypertension, diabetes, dyslipidemia, and other general characters such as smoking and drinking (11, 14–16). Most of them were in accordance with our results, and in our study, hypertension caused ICH more in HHcy group than in Hcy normal group, which indirectly illustrated the relationship between Hcy and atherosclerotic risk factors. These factors and unhealthy habits, such as cigarettes smoking, may interfere the absorption of vitamin B6, B12, and folate and increase the Hcy level and formation of atherosclerosis (17). In our study, there were only 165 female patients included. Since previous studies showed that women always had a less favorable prognosis of stroke than men, and post-menopausal women with high Hcy had a higher relative risk of cardiovascular events, we assumed that the estrogen level could regulate the Hcy concentration and made women more susceptible to the pathogenic processes after the ICH onset (16, 18, 19).

The pathogenesis of HHcy in cardiovascular and cerebrovascular diseases varies a lot. Previous studies demonstrated that HHcy could increase the production of hydrogen peroxide and decrease the activity of nitric oxide synthase, DNA methylation, and fibroblast growth factors, affecting the endothelial cell diastolic function and apoptosis. The hyalinization and apoptosis of the endothelial cells on small artery could then cause ICH and influence its prognosis (11, 20, 21). The dyslipidemia mechanism reported that HHcy may accelerate the generation of reactive oxygen species on endothelial cells, and more foam cells were formed to the atherosclerotic change (9, 22). What is more, HHcy could affect the L-arginine/nitric oxide pathway of platelet and break the balance of coagulation and fibrinolysis system. To the inflammatory reaction and oxidative stress theory, HHcy may accelerate the generation of high-sensitivity C-reactive protein, interleukin-8, caspase-9, matrix metalloproteinase, and other proteins to participate the early-state formation of atherosclerosis. The cumulation of inflammatory factors could then accelerate the progress of ICH (23–26). We therefore infer Hcy may aggravate the process of atherosclerosis and elevate the risk of hemorrhagic vascular diseases by the coefficient results of the above pathogenesis we mentioned (9).

Potential limitations of our study should be discussed. First, the patients were all from Beijing, northern China. A similar dietary habit or some specific genetic diseases may influence the level of Hcy, so some bias may exist because of the population. Second, during the study, we did not collect the concentration of vitamin and folate among patients and the therapeutic method of HHcy were also not recorded during the follow-up. These may influence part of the analysis about the pathogenesis of Hcy on ICH prognosis. Third, we only collected the Hcy level at the acute phase of ICH but did not collect it prior to the onset of ICH or during the follow-ups. Therefore, it is unclear what the potential effects of the acute stress reaction of ICH may have had on the Hcy levels measured subsequent to ICH onset. So, after we found out the positive relationship in this study, we will try to continue exploring how Hcy influences the whole process of ICH and the pathogenesis between Hcy and ICH with more particular and specific indexes dynamically and more diverse racial populations. Meanwhile, we will explore whether lowering the level of Hcy could decrease the morbidity or mortality of ICH and try to find a new therapeutic or preventive way of managing ICH in the future.

## Summary

In conclusion, the present study showed high Hcy level to be independently associated with poorer 3 month prognosis and a lower survival rate within 1 year in patients with ICH. These results suggest the possibility that in patients with acute ICH, high Hcy concentration plays a role in the development of and recovery after ICH onset.

## Data Availability Statement

The raw data supporting the conclusions of this article will be made available by the authors, without undue reservation.

## Ethics Statement

Written informed consent was obtained from the individual(s) for the publication of any potentially identifiable images or data included in this article.

## Author Contributions

DW analyzed and interpreted the data and drafted the manuscript. WW and XZ conceived and designed the research. XZ handled funding and supervision. AW acquired the data. All authors contributed to the article and approved the submitted version.

## Conflict of Interest

The authors declare that the research was conducted in the absence of any commercial or financial relationships that could be construed as a potential conflict of interest.

## Abbreviations

ICH: intracerebral hemorrhage
Hcy: homocysteine
IRB: Institutional Review Board
CT: computerized tomography
HHcy: high homocysteine
FBG: fasting blood glucose
HbA1c: hemoglobin A1c
TC: total cholesterol
TG: triglyceride
LDL-C: low-density lipoprotein cholesterol
HDL-C: high-density lipoprotein cholesterol
Hs-CRP: high-sensitivity C-reactive protein
BMI: body mass index
CAA: cerebral amyloid angiopathy
NIHSS: National Institute of Health Stroke Scale
GCS: Glasgow Coma Scale
mRS: modified Rankin Scale
OR: odd ratio
CI: confidence interval.
