# Supplementary material for: Association of Severity and Prognosis With Elevated Homocysteine Levels in Patients With Intracerebral Hemorrhage
Source: Front Neurol. 2020 Oct 19;11:571585. doi: 10.3389/fneur.2020.571585 (PMC7604273; doi:10.3389/fneur.2020.571585)
Supplement: Supplementary file 1 [file Table_1.docx]

Supplementary Table 1. Baseline characteristics and their univariate between included and excluded participants

|  | included  n=551 | excluded  n=1413 | P value |
| --- | --- | --- | --- |
| Male– no.(%) | 386 (70.1) | 941 (66.6) | 0.1477 |
| Age (IQR) -yr | 58.0 (49.0, 67.0) | 56.0 (48.0, 66.0) | 0.2188 |
| BMI (IQR) | 25.2 (22.9, 27.6) | 25.4 (22.9, 27.7) | 0.7896 |
| Smoking– no.(%) | 184 (33.4) | 444 (31.4) | 0.4193 |
| Drinking– no.(%) | 240 (43.6) | 476 (33.7) | <0.0001 |
| Previous mRS (IQR) | 0 (0, 0) | 0 (0, 0) | 0.4942 |
| SBP (IQR) -mmHg | 160.0 (143.0, 179.0) | 169.0 (149.0, 190.0) | <0.0001 |
| DBP (IQR) -mmHg | 92.0 (80.0, 105.0) | 97.0 (83.0, 110.0) | 0.0033 |
| FBG (IQR) -mmol/l | 5.5 (4.6, 6.7) | 6.2 (5.1, 7.9) | <0.0001 |
| HbA1c– no.(%) | 5.6 (5.2, 6.1) | 5.6 (5.3, 6.2) | 0.2778 |
| TC (IQR) -mmol/l | 4.5 (3.9, 5.3) | 4.4 (3.7, 5.1) | 0.0047 |
| TG (IQR) -mmol/l | 1.2 (0.9, 1.7) | 1.2 (0.8, 1.8) | 0.1986 |
| LDL-C (IQR) -mmol/l | 2.9 (2.3, 3.5) | 2.6 (2.1, 3.3) | <0.0001 |
| HDL-C (IQR) -mmol/l | 1.2 (1.0, 1.5) | 1.2 (1.0, 1.5) | 0.1003 |
| Hs-CRP (IQR) -mg/l | 4.9 (1.8, 11.5) | 6.4 (1.9, 12.0) | 0.4053 |
| Complicating disease– no.(%) |  |  |  |
| Hypertension | 373 (67.7) | 873 (61.8) | 0.0164 |
| DM | 87 (15.8) | 175 (12.4) | 0.0546 |
| Dyslipidemia | 204 (37.0) | 188 (13.3) | <0.0001 |
| PI | 122 (22.1) | 453 (32.1) | <0.0001 |
| DVT | 55 (10.0) | 67 (4.7) | <0.0001 |
| AF | 10 (1.8) | 23 (1.6) | 0.8452 |

IQR: interquartile range, BMI: Body Mass Index, mRS: modified Rankin Scale, SBP: Systolic blood pressure, DBP: Diastolic blood pressure, FBG: fasting blood glucose, HbA1c: hemoglobin A1c, TC: total cholesterol, TG: triglyceride, LDL-C: low-density lipoprotein cholesterol, HDL-C: high-density lipoprotein cholesterol, Hs-CRP: High- sensitivity C-reactive protein, DM: Diabetes Mellitus, PI: Pulmonary infection, DVT: Deep vein thrombosis, AF: Atrial fibrillation
